# Rhein, a novel Histone Deacetylase (HDAC) inhibitor with antifibrotic potency in human myocardial fibrosis

**DOI:** 10.1038/s41598-020-61886-3

**Published:** 2020-03-17

**Authors:** David Monteiro Barbosa, Pia Fahlbusch, Daniella Herzfeld de Wiza, Sylvia Jacob, Ulrike Kettel, Hadi Al-Hasani, Martina Krüger, D. Margriet Ouwens, Sonja Hartwig, Stefan Lehr, Jorg Kotzka, Birgit Knebel

**Affiliations:** 1 Institute of Clinical Biochemistry and Pathobiochemistry, German Diabetes Center at the Heinrich-Heine-University Duesseldorf, Leibniz Center for Diabetes Research, Aufm Hennekamp 65, 40225 Duesseldorf, Germany; 2 German Center for Diabetes Research (DZD), Munich-Neuherberg, Germany; 3 Medical Faculty, Institute of Cardiovascular Physiology, Heinrich-Heine-University, Duesseldorf, Germany; 4 Medical Faculty, Institute for Clinical Biochemistry and Pathobiochemistry, German Diabetes Center (DDZ), Heinrich-Heine-University, Duesseldorf, Germany; 5 Department of Endocrinology, Ghent University Hospital, Ghent, Belgium

**Keywords:** Cardiology, Molecular medicine

## Abstract

Although fibrosis depicts a reparative mechanism, maladaptation of the heart due to excessive production of extracellular matrix accelerates cardiac dysfunction. The anthraquinone Rhein was examined for its anti-fibrotic potency to mitigate cardiac fibroblast-to-myofibroblast transition (FMT). Primary human ventricular cardiac fibroblasts were subjected to hypoxia and characterized with proteomics, transcriptomics and cell functional techniques. Knowledge based analyses of the omics data revealed a modulation of fibrosis-associated pathways and cell cycle due to Rhein administration during hypoxia, whereas p53 and p21 were identified as upstream regulators involved in the manifestation of cardiac fibroblast phenotypes. Mechanistically, Rhein acts inhibitory on HDAC classes I/II as enzymatic inhibitor. Rhein-mediated cellular effects were linked to the histone deacetylase (HDAC)-dependent protein stabilization of p53 under normoxic but not hypoxic conditions. Functionally, Rhein inhibited collagen contraction, indicating anti-fibrotic property in cardiac remodeling. This was accompanied by increased abundance of SMAD7, but not SMAD2/3, and consistently SMAD-specific E3 ubiquitin ligase SMURF2. In conclusion, this study identifies Rhein as a novel potent direct HDAC inhibitor that may contribute to the treatment of cardiac fibrosis as anti-fibrotic agent. As readily available drug with approved safety, Rhein constitutes a promising potential therapeutic approach in the supplemental and protective intervention of cardiac fibrosis.

## Introduction

Heart failure (HF) accounts for the most deaths in both first- as well as third-world countries^1^. A hallmark of HF is adverse cardiac remodeling following cardiac injury, which is characterized by dysregulated fibrosis^2^. During cardiac fibrosis an imbalance between extracellular matrix (ECM) turnover and synthesis results in the formation of excess fibrous connective tissue^3^. This accumulation of ECM in the left ventricle is directly associated with increased mechanical stiffness, hence contributing to both systolic and diastolic dysfunction^4^. A key process in cardiac fibrosis is the differentiation of resident cardiac fibroblasts (CFs) to myofibroblasts (MFs)^5^, also referred to as fibroblast-to-myofibroblast transition (FMT). Switching to the MF phenotype is characterized by tremendously increased motility, migration and adhesion^6^ and by gain of contractile function due to the *de novo* formation of α-smooth muscle actin (αSMA) fibers, important for wound closure and structural integrity during scar formation^7^. Although FMT constitutes an essential reparative and compensatory mechanism, persistent myofibroblast activation may lead to the dissemination of fibrotic areas from the point of lesion towards the remote interstitium, accelerating HF^8^.

Characteristically, fibrosis-associated diseases feature chronic tissue hypoxia, caused by microvascular obstruction and high oxygen consumption by resident cells^9^ or infiltrating inflammatory and mesenchymal cells^10^. Interestingly, chronic or prolonged periods of hypoxia have been hypothesized to be involved in adverse fibrosis, directly associated to changes of fibroblast behavior and modulation of secreted soluble factors i.e. VEGF or TGFβ by CFs^8,11,12^. Thus, the local intercellular crosstalk through different secretion profiles constitutes a possible driving mechanism in the propagation of MFs throughout the heart and for maladapted remodeling. Targeting the cardiac MF secretome is therefore a proposed area of interest to develop anti-fibrotic therapies and promote cardiac preservation in HF patients^11^.

Rhein (4,5-dihydroxyanthraquinone-2-carboxylic acid), a lipophilic anthraquinone, is a pharmaceutically active component predominantly found in rhubarb (*Rheum palmatum* L.), and extensively used in traditional Chinese medicine^13^. Since the early 1990s, Rhein has found clinical appreciation in the treatment of osteoarthritis by decreasing inflammation and cartilage destruction^14^. Based on the wide potential applicability^13^, repurposing of Rhein as treatment for several other pathologies besides osteoarthritis has been considered. Interestingly, increasing evidence suggests Rhein as potential anti-fibrotic agent in several organs^15–17^. The role of Rhein in cardiac pathology has been scarcely investigated, yet one study reported that administration of its pro-drug form Diacerein improved left ventricular remodeling and cardiac function after myocardial infarction in rats^18^.

Still, the exact mechanism of how Rhein as small molecule interferes with the progression of fibrosis has not been sufficiently addressed. Therefore, in this study the effect of Rhein administration on FMT of hypoxia-treated primary human ventricular cardiac fibroblasts (HCF-v) was investigated.

## Results

### The impact of Rhein on chronic hypoxia-mediated modulation of HCF-v phenotype and secretory profiles

**Figure 1:**
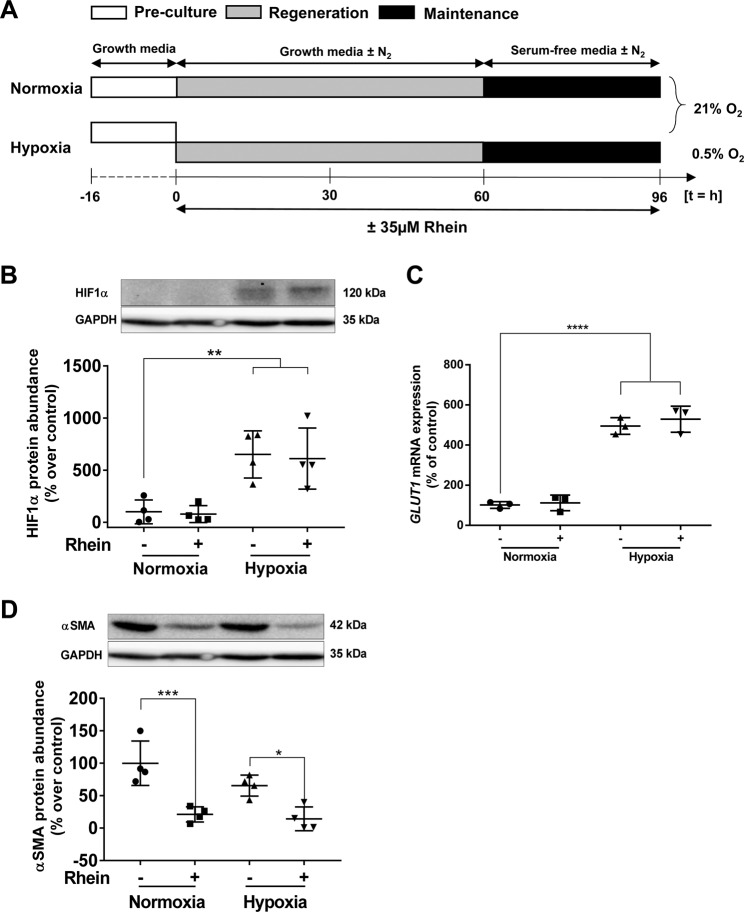
Rhein reduces αSMA protein abundance independently from oxygen. (**A**) Experimental setup of hypoxia treatment. HCF-v were adhered for 16 h, grown for 60 h under normoxic (21% O_2_) or hypoxic (0.5% O_2_) conditions and subsequently serum-starved for 36 h. With every change of the media (every 30 h), respective cells were treated with 35 µM Rhein throughout the duration of the experiment (96 h). (**B**) Rhein does not affect HIF1α stabilization. Representative blot and quantification of HIF1α abundance (n = 4). (**C**) mRNA expression of the direct HIF1α target *Glut1*. (**D**) Rhein-mediated αSMA protein reduction. Representative Western blot and quantification of relative protein abundance (n = 4). Antibodies in (**B**,**D**) were probed to the same membrane. All data are presented as mean ± SD. One-way-ANOVA with post-hoc Sidak’s multiple comparison, *p < 0.05, **p < 0.01, ***p < 0.001, as indicated.

To study the potential effect of Rhein on phenotype and secretome modulation, cells were exposed for 4d to normoxic or hypoxic conditions (Fig. 1A) in the absence or presence of a non-toxic concentration of 35 µM Rhein (Supplementary Fig. 1). The hypoxic treatment protocol was monitored by analyses of hypoxia marker HIF1α and its direct transcriptional downstream target *GLUT1*, which revealed an approximate 6-fold increased protein abundance and 5-fold increased transcription in hypoxia exposed cells (Fig. 1B,C), respectively. Rhein treatment did not further affect HIF1α abundance neither under normoxic nor hypoxic environment. In the presence of Rhein, cells contained substantially decreased αSMA protein amounts independently of whether exposed to normoxia (80% reduction, p < 0.001) or hypoxia (85% reduction, p < 0.05) (Fig. 1D). Furthermore, Rhein treatment showed an overall reduction in the expression of exemplary pro-fibrotic response targets (Supplementary Fig. 2A; *ACTA2, CTGF, COL1A1, COL3A1, OGN, ITGA8, TIMP3*) and increased *MMP1* expression pointing towards a higher MMP:TIMP ratio.

Secretome analyses identified a total of 2,168 secreted proteins. The comparison of normoxic and hypoxic conditions (NvsH) revealed 638 differentially abundant proteins (>1.5-fold, p < 0.05), including 95 uniquely present proteins, whereas 619 were found in normoxic versus hypoxic cells both treated with Rhein (NRvsHR) including 46 uniques, 1,010 proteins in normoxic versus Rhein-treated normoxic cells (NvsNR) with unique 170 and overall 590 differentially abundant proteins with 74 solely observed in the comparison of hypoxic and Rhein-treated hypoxic cells (HvsHR), respectively (Supplementary Table 1).

**Figure 2:**
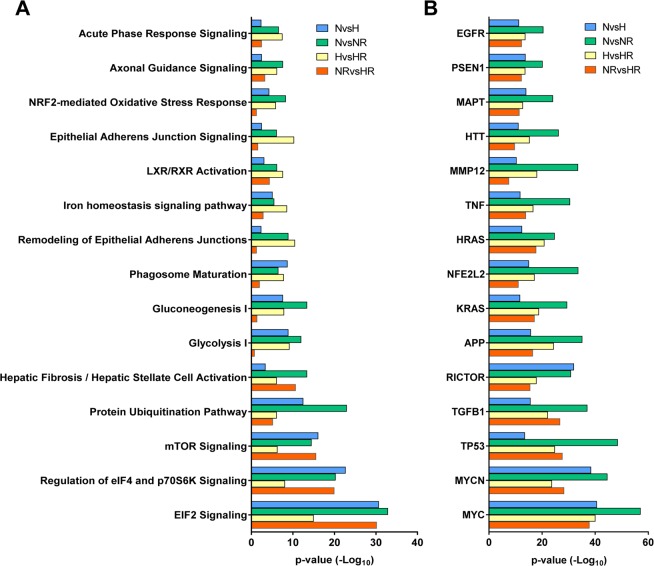
Effect of Rhein on the secretome of cardiac fibroblast under normoxic and hypoxic environments. (**A**) Graph showing the global top 15 annotated canonical pathways of differential secretomes sorted by cumulative −Log_10_ p-value. (**B**) Graph showing the global top 15 predicted upstream regulators of differential secretomes sorted by cumulative −Log_10_ p-value. The graphs were generated through the use of IPA (QIAGEN Inc., https://www.qiagenbioinformatics.com/products/ingenuity-pathway-analysis).

To explore the global effect of hypoxia and Rhein intervention on secretome modulation, here, we focused on commonly annotated pathways responsible for all differentially abundant proteins within each comparison. Among the top 15 pathways (Fig. 2A), highest cumulative significance was found in canonical pathways associated to ER stress and translational regulation (EIF2 signaling (−log_10_ p = ∑108.8)), Regulation of eIF4 and p70S6K Signaling (∑71.1), mTOR signaling (∑52.5) and Protein Ubiquitination Pathway (∑46.8)). Further, robust enrichment was identified in pathways related to ECM modulation and Fibrosis (Hepatic Fibrosis/Hepatic Stellate Cell Activation (−log_10_ p = ∑33.7)), Epithelial Adherens Junctions Remodeling (∑23.2) and Signaling (∑20.5), or Axonal Guidance Signaling (∑19.5).

Within the top 15 upstream regulators predicted to be causative in the condition-dependent secretomes (Fig. 2B), cell fate- and cell cycle-determining MYC (−log_10_ p = ∑175.8), MYCN (∑135.1) and TP53 (∑114.7) constituted the most significant upstream regulators in the context of hypoxia and intervention via Rhein administration.

### Influence of Rhein administration on transcriptomic profiles in cardiac fibroblasts under normoxic and hypoxic conditions

**Figure 3:**
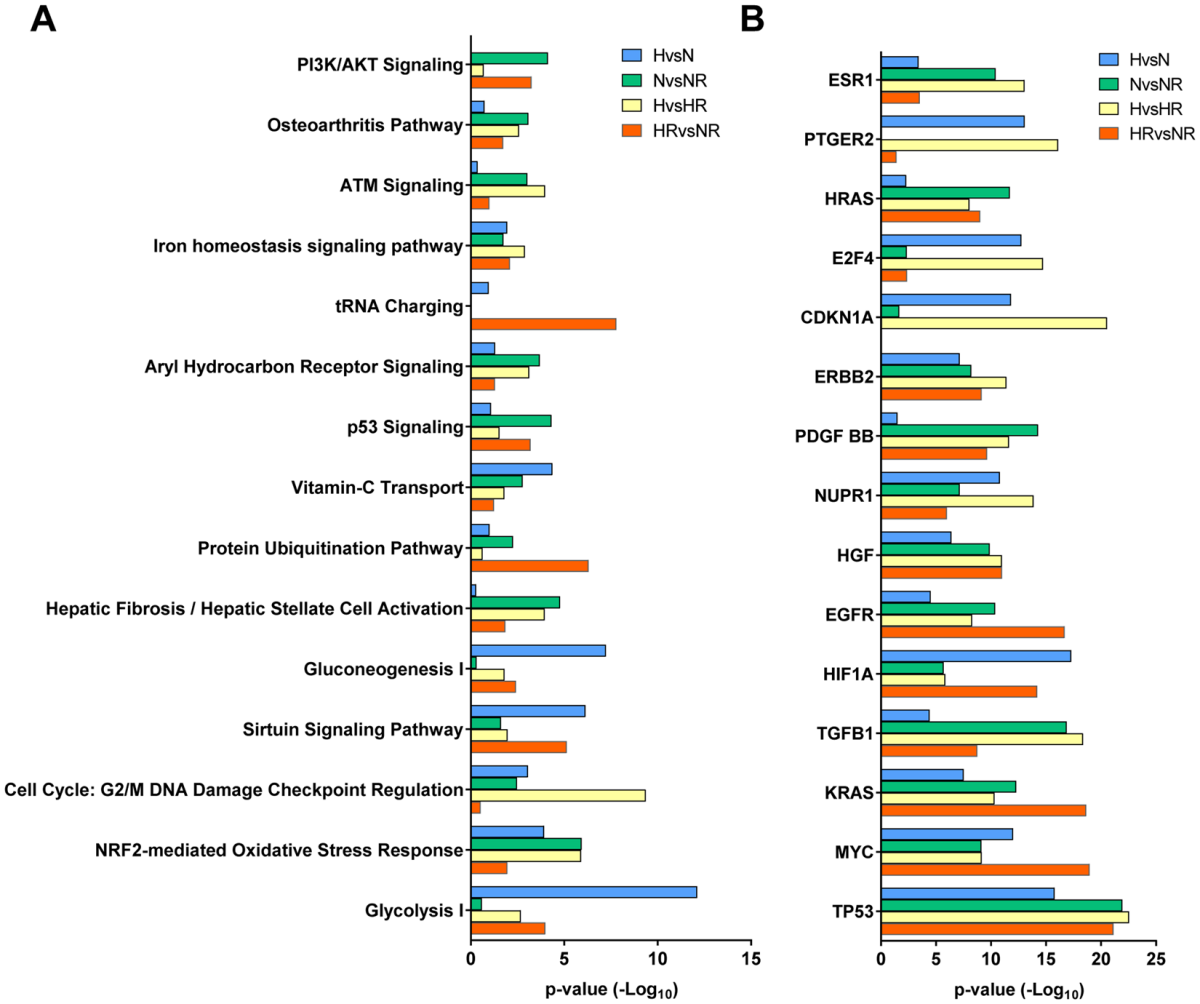
Influence of Rhein administration on transcriptomic profiles in cardiac fibroblasts under normoxic and hypoxic conditions. (**A**) Graph showing the global top 15 canonical pathway annotations of differential transcripts sorted by cumulative −Log_10_ p-value. (**B**) Graph showing the global top 15 predicted upstream regulators of differential transcripts sorted by cumulative −Log_10_ p-value. N: Normoxia, H: Hypoxia, NR: Normoxia + Rhein, HR: Hypoxia + Rhein.

To further dissect how Rhein treatment is able to modulate the secretome of HCF-v, the impact of Rhein on RNA expression was examined under the identical experimental conditions. Here, we identified 136 out of 325 differentially regulated transcripts (>1.5-fold, p < 0.05) solely found in HvsN, while the comparison HRvsNR harbored 719 out of 2,175 transcripts uniquely expressed. In comparison to that, 1,210 out of 2,916 mRNAs and 247 out of 803 transcripts were exceptionally observed within the group of NvsNR and HvsHR, respectively. Analogously to the secretome analyses, all differentially abundant transcripts within each assigned comparison were examined for commonly annotated pathways. Here we found overlaps in the regulation of similar pathways, specifically in the groups NvsNR and HvsHR associating to fibrosis-related pathways like Hepatic Fibrosis/Hepatic Stellate Cell Activation (−log_10_ p-value = 4.8 for NvsNR/−log_10_ p-value = 4.0 for HvsHR) and Osteoarthritis Pathway (3.1/2.6) (Fig. 3A). Most interestingly, transcriptome analysis revealed an enrichment of cell cycle pathway annotations (Cell Cycle: G2/M DNA Damage Checkpoint Regulation (−log_10_ p = ∑15.4) and p53 Signaling (∑10.1)). In accordance to the identification of TP53 as potential upstream regulator of the secretome, we also identified TP53 (−log_10_ p = ∑81.4) as top affected target responsible for transcriptomic changes in all 4 groups (Fig. 3B). Moreover, CDKN1A (−log_10_ p = ∑34.0), an imminent target of p53 transcription factor, was identified to be significantly affected in all comparison groups with exception of HRvsNR.

### Rhein identifies as HDAC inhibitor and affects cell cycle regulators p53 and p21

The expression status of known positive and negative regulators of the G2/M phase derived from the overall gene expression analyses pointed towards an impact of cell cycle progression in Rhein-mediated phenotype modulation (Supplementary Fig. 2B). Here, in HCF-v subjected to hypoxia (H) increased expression of negative regulators like PLK1, CCNA2, CDK1 or CCNB2, while positive regulators were not affected. Rhein administration (HR) abolished activation of negative regulators, but downregulated positive regulators e.g. BRCA2 and ATR. Administration of Rhein under normoxic conditions (NR) seemed to favor expression of positive regulators (RAD9A, HUS1, or PALB2).

**Figure 4:**
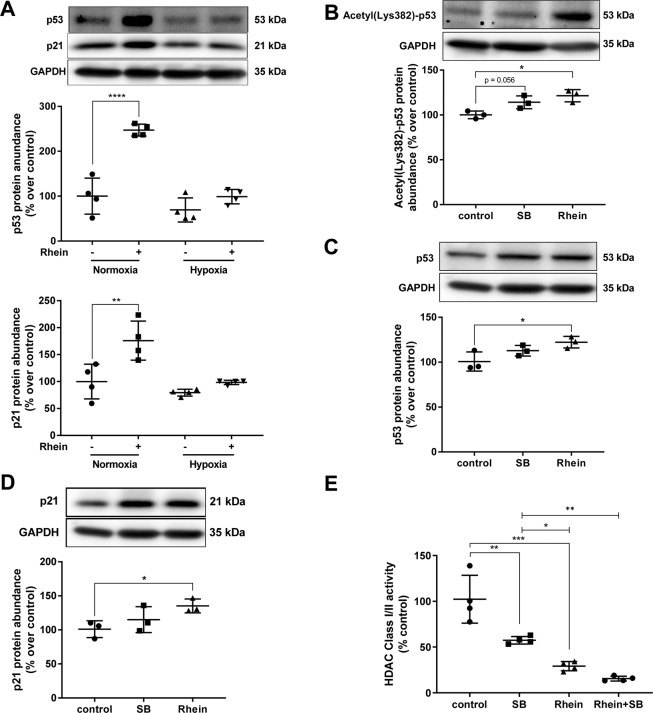
Effect of Rhein on HDAC activity and G2/M cell cycle phase regulation, p53 and p21 abundance and proliferation markers. (**A**) Representative blots and quantification showing Rhein-mediated increase of p53 and p21 abundance (n = 4). (**B**) Representative blots and quantification showing Rhein-driven p53 stabilization by increased HDAC inhibition-mediated acetylation at Lys382 (n = 4). 1 mM sodium butyrate (SB) was used as reference HDAC inhibitor. (**C**) Representative blots and quantification showing Rhein-mediated increase of p53 abundance (n = 4) under conditions described in (**B**). (**D**) Representative blots and quantification showing Rhein-mediated increase of p21 abundance (n = 4) under conditions described in (**B**). Antibodies in (**C**,**D**) were probed to the same membrane. (**E**) Enzymatic HDAC activity is inhibited in the presence of Rhein. Graph showing HDAC activity after 30 min treatment of normal cell lysates with Rhein or SB (n = 4). All data are presented as mean ± SD. One-way-ANOVA with post-hoc Sidak’s multiple comparison, *p < 0.05, **p < 0.01, ***p < 0.001, as indicated.

**Figure 5:**
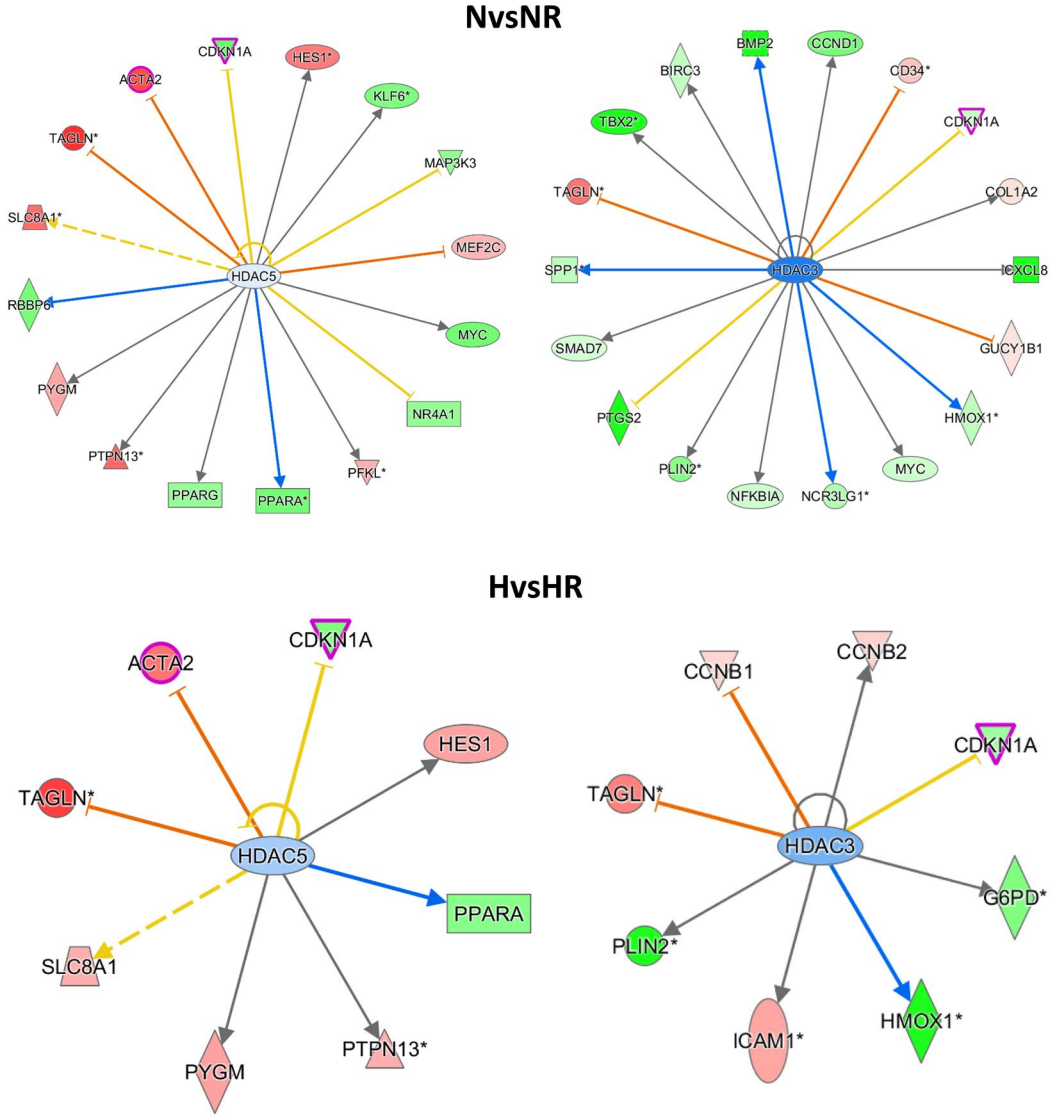
HDAC3/5 regulated downstream targets in transcriptome analyses. Rhein mediated effects were analyzed in the comparison groups NvsNR and HvsHR. Fold changes of up- and down-regulated genes are scaled in shades of red (higher expression in first condition of comparison) and green (lower in first condition), respectively. Blue color of upstream target indicates predicted inhibition. Orange and blue arrows indicate indirect activation and inhibition; yellow and gray arrows represent inconsistent effects and no prediction, respectively. The networks were generated through the use of IPA (QIAGEN Inc., https://www.qiagenbioinformatics.com/products/ingenuity-pathway-analysis).

To verify observations of differential gene expression analyses, abundances of predicted upstream regulators p53 and p21 (encoded by *CDKN1A*) were determined. Following Rhein treatment both p53 and p21 protein abundances were significantly increased under normoxic conditions by 2.5-fold (p < 0.0001) and 1.7-fold (p < 0.01), respectively, but was not significantly different under hypoxic conditions (Fig. 4A). While *CDKN1A* was induced and differentially regulated on transcriptional level following Rhein treatment (Supplementary Fig. 2A), intriguingly, TP53 was not differentially regulated, indicating a posttranslational mechanism leading to increased p53 abundance. We tested whether Rhein affected p53 acetylation, a modification leading to increased protein stability, in analogy to HDAC inhibition-mediated effects. HDAC inhibition via sodium butyrate (SB) showed a trend (p = 0.056) towards increased p53 acetylation, whereas Rhein treatment moderately but significantly enhanced p53 acetylation (1.2-fold; p < 0.05) (Fig. 4B). In parallel, SB-mediated HDAC inhibition only showed a trend towards higher presence of p53 protein abundance, while Rhein treatment significantly increased (1.2-fold, p < 0.05) p53 protein (Fig. 4B,C). In accordance, nodal cell cycle stages of G1/S and G2/M transition were differentially regulated in the comparisons (Supplementary Fig. 3). In addition, HDAC inhibition via SB or Rhein also revealed a trend towards higher p21 abundance (Fig. 4D), which seemed proportional to increased p53 levels. Surprisingly, HDAC expression levels derived from holistic gene expression analyses throughout all members of the HDAC family was unchanged (Supplementary Fig. 2C). Hence, we analyzed whether Rhein imminently affected HDAC activity by direct enzymatic inhibition. We performed these experiments independent from cellular context in cell lysates. As a key finding, Rhein significantly inhibited deacetylase activity by 70% (p < 0.001), which even was superior to the SB-mediated HDAC inhibition by 43% (p < 0.01) (Fig. 4E). In further detail, the direct addition of Rhein to the cell lysates is necessary to interfere with HDAC activity, independent to prior growth conditions. Rhein then is a more efficient HDAC enzymatic activity inhibitor than the commonly used inhibitors in a direct mode that even additive specifically in HDAC class I (Supplement Fig. 4), or SIRT (HDAC class III) (Supplement Fig. 5). The interrelation of HDAC and CDKN1A in the focus of Rhein treatment was also found by the examples of HDAC3 and HDAC5 upstream regulator analyses. According to IPA^®^, *CDKN1A* is negatively regulated by HDAC3/5, which matched the observed decreased *CDKN1A* mRNA expression in the comparisons NvsNR and HvsHR (Fig. 5). Of further notice, HDAC5 seemed to be associated with FMT marker *ACTA2*, which we observed to be downregulated by Rhein.

### Rhein administration blocks TGFβ1-stimulated FMT on molecular and functional level

**Figure 6:**
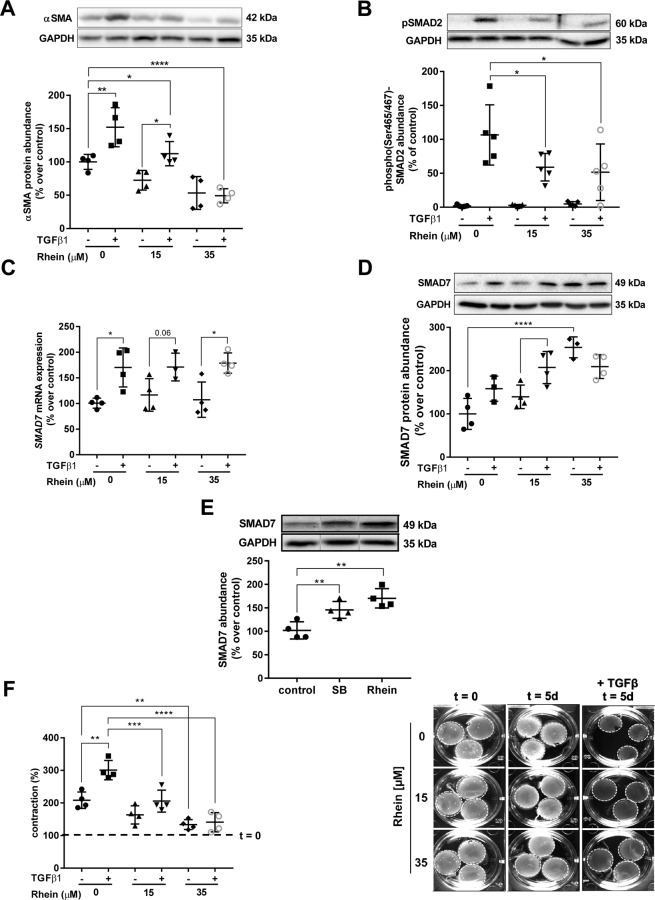
Rhein functionally inhibits TGFβ1-stimulated FMT. (**A**) Representative Western Blot and quantification of αSMA relative protein abundance (n = 4). (**B**) Rhein inhibition of TGFβ1/SMAD signaling. Representative blot and quantification of phospho(Ser465/467)-SMAD2 abundance in Rhein and TGFβ1 treated cells. (**C**) Expression analysis of *SMAD7* showing TGFβ1-mediated increase of transcription and absence of Rhein-mediated effects (n = 4). (**D**) Rhein increases SMAD7 abundance independently from TGFβ1. Representative blot and quantification showing increased basal SMAD7 abundance after Rhein treatment and TGFβ1-dependent SMAD7 expression (n = 4). (**E**) Effect of HDAC inhibition on SMAD7 stabilization. Representative blot and quantification showing increased protein abundance after Rhein and SB treatment (N = 4). (**F**) FPCL contraction assay. Quantitative analysis on the contraction of experimental FPCLs (n = 4). Representative overhead pictures of FPCLs before (d0) and after treatment (d0) with TGFβ1 alone or in combination with Rhein. Dashed line indicates baseline (start point at d0). All data are presented as mean ± SD. One-way-ANOVA with post-hoc Sidak’s multiple comparison, *p < 0.05, **p < 0.01, ***p < 0.001, ****p < 0.0001 as indicated.

Next, as proof of principle we studied the anti-fibrotic properties of Rhein in the setting of TGFβ1-induced FMT. Dose-response analyses revealed a concentration-dependent effect of Rhein on selected cardiac fibrosis-relevant proteins and mRNAs (Supplementary Fig. 6A–C). Consequently, to evaluate whether Rhein affects FMT, cells were therefore exposed to 15 µM or 35 µM Rhein alone or in combination with TGFβ1 for 24 h (Fig. 6A–D). Consistent with the dose-dependent reduction of TGFβ1-promoted induction of *ACTA2* (Supplementary Fig. 6D) Rhein also decreased the protein abundance of αSMA (Fig. 6C; 15 µM: 113%, p < 0.05; 35 µM: 49%, p < 0.0001). However, TGFβ1 target SMAD3 were not affected by Rhein on protein or mRNA level (Supplementary Fig. 7A–D). Further, 35 µM Rhein completely abrogated the response of cells to TGFβ1-induced accumulation of αSMA. HCF-v pre-treated with Rhein for 24 h prior to 30 min incubation with TGFβ1, demonstrated decreased abundance and expression of TGFβ receptor I (Supplementary Fig. 6E,F) along with significantly decreased phosphorylation of SMAD2 at Ser465/467 by 50% (p < 0.05) (Fig. 6B, Supplementary Fig. 7E). Stimulation of HCF-v with TGFβ1 expectedly led to transcriptional activation of *SMAD7* (p < 0.01, Fig. 6C) and increased protein abundance (p < 0.05, Fig. 6D), as part of the TGFβ/SMAD7 negative feedback loop. Most interestingly, a Rhein-mediated dose-dependent SMAD7 protein accumulation in TGFβ1-naive cells was observed, displaying 2.5-fold higher protein levels in 35 µM Rhein treated cells (p < 0.0001). However, this effect was not transcription-dependent, again pointing towards a role of posttranslational mechanisms. Indeed, HDAC inhibition via SB for 24 h increased SMAD7 protein abundance by 1.5-fold (p < 0.01) and 1.7-fold after Rhein administration (p < 0.01) (Fig. 6E) proportionally to the degree of HDAC activity inhibition, as determined before. In line, SMAD-specific E3 ubiquitin ligase SMURF2 but not SMURF1 protein stability increased in parallel (Supplementary Fig. 8A–D). Finally, to evaluate whether Rhein also functionally inhibited FMT, fibroblast-populated collagen lattice (FPCL) contraction assays were performed. Rhein treatment of FPCLs confirmed molecular observations on cell contractility (Fig. 6F), showing that TGFβ1-induced contraction was dose-dependent decreased by Rhein (p < 0.001). Of further notice, TGFβ1-mediated contraction in relation to respective unstimulated control was reduced upon administration of 15 µM (163% versus 208% to baseline) and 35 µM Rhein (133% versus 141%). Most surprisingly, we also found that contraction in TGFβ1-naive samples (208% to baseline) was significantly (p < 0.001) decreased in cells exposed to 35 µM Rhein (133% to baseline), suggesting a protective effect of Rhein on overall FMT.

## Discussion

The purpose of this study was to examine the potential role of the rhubarb anthraquinone Rhein as potential anti-fibrotic agent in the setting of cardiac fibrosis. To address this question, an *in vitro* model of sustained hypoxia as pathophysiological mediator of a pro-fibrotic environment in primary HCF-v was employed. In the present study, following chronic hypoxia, cellular HIF1α protein abundance was significantly increased. This is in line with common observations as HIF1α is regarded the key regulator of adaptation to hypoxic environment^19^. Further, knowledge-based analyses of the transcriptome predicted the regulation of several hypoxia-associated metabolic pathways such as glycolysis and gluconeogenesis. Together, these findings prove that cells were indeed experiencing hypoxia verifying the validity of the presently employed protocol.

Several studies indicated that both acute (24–72 h) and prolonged hypoxia (4–8d) directly initiated FMT marked by pronounced αSMA expression^12–14,20–22^.

In this study, chronic hypoxia did not affect αSMA protein abundance, which could be the result of the employment of different O_2_ tension or culture conditions (media, cell density, etc.). However, aside of αSMA as main marker of mature myofibroblasts, hypoxia affected representative pro-fibrotic genes including cytokines, proteoglycans, integrins, MMPs and their inhibitors TIMPs, indicating increased myofibroblast activity^23^. The key finding that Rhein substantially reduced αSMA protein abundance in HCF-v and oppositely regulated other pro-fibrotic genes was in line with several observations reported in other tissues^24–26^.

Pronounced pathological dissemination of myofibroblasts and adverse fibrosis have been associated to changes in fibroblast secretory profiles^8,11^. Thus, we screened for common differentially regulated pathways and upstream regulators between all secretomes to determine possible factors that could be responsible for the orchestration of Rhein-mediated effects. Here, we identified pathways associated to ER stress, translational regulation, as well as ECM remodeling to be crucial in the modulation of respective secretory profiles. Global transcriptomic analyses partly confirmed pathways identified in the secretome, presenting overlaps in regulated pathways. In addition, apart from high coverage of predicted pathways, transcriptome analyses uniquely revealed a role of cell cycle and regulatory proliferation pathways to significantly participate in the phenotype modulation. Consistently, both secretome and transcriptome analyses commonly identified p53 as confident affected upstream regulator. This finding supports already published work, showing the interaction between Rhein and p53^27,28^, collectively proving the functionality of Rhein and its action in the presently applied system. Furthermore, computational analysis also predicted *CDKN1A* (p21) as potential candidate, an imminent downstream target of p53 and known key regulator of proliferation and cell cycle^29,30^.

Proliferation of myofibroblasts is a key feature of wound healing and adverse fibrosis^6,31^. In the present study, we observed upregulated levels of proliferation marker *MKI67*^32,33^ under hypoxic conditions, consistent with other reports, showing increased hypoxia-mediated proliferation of cardiac^20^ and pulmonary fibroblasts^34^. The strongly reduced expression of *MKI67* ascribed Rhein a novel anti-proliferative property in regard to cardiac fibrosis. Cell cycle regulation at the level of G2/M checkpoint has been shown to be modulated by hypoxia^35^. Consistently, we also determined upregulated expression of negative regulators in our approach, pointing towards abrogated G2/M checkpoint regulation. Intriguingly, after Rhein treatment we observed differential regulation of negative and positive regulators, indicating a prolonged G2/M phase. Most interestingly, Rhein induced both expression and protein levels of cell cycle arrest inducer *CDKN1A* (p21), in line with a previous report, where Rhein-mediated suppression of proliferation in chondrocytes associated to increased p21 levels^36^. Although p53 was multiply predicted as robust upstream regulator throughout all conditions, *TP53* mRNA levels were not affected in any experimental group. However surprisingly, the presence of Rhein under normoxic conditions increased p53 protein levels, in line with other reports^27,28,37^. Collectively, these findings pointed towards a posttranslational effect of Rhein on p53, leading to its increased abundance and transcriptional activity. Regulation of gene transcription also occurs via acetylation of non-histone proteins, like transcriptional factors, by affecting their DNA binding affinity^38^. Further, acetylation of lysine residues competitively blocks them for ubiquitin ligases, protecting proteins from ubiquitination and subsequent degradation by the proteasome^39^. Eventually, it has been reported that HDAC inhibition-mediated hyperacetylation and stabilization of p53 at lysine residue K382 increased p53-dependent p21 expression^40–42^. In the present study, application of Rhein significantly increased both p21 protein abundance and p53 acetylation at K382 comparable to effects of HDAC inhibition using the reference inhibitor SB. Of utmost interest, in functional HDAC activity assays we could show that Rhein strongly inhibited HDAC Class I/II, providing first-hand evidence identifying Rhein as novel HDAC inhibitor. Supported by the observation that expression of HDACs was unchanged, thus, we propose that Rhein might act as imminent and reversible inhibitor. Interestingly, exemplary analysis of HDAC3/5 as upstream regulators in our transcriptomic data set not only confirmed the connection between HDAC and *CDKN1A*, but also demonstrated a link between HDAC and *ACTA2*. Indeed, it was shown that the pan-HDAC inhibitor trichostatin A blocks αSMA induction associated with reduced activation of Akt in lung fibroblasts^43^ and inhibits TGFβ1-induced collagen synthesis in cultured rat cardiac fibroblasts. Further, administration of MGCD0103 even demonstrated reversion of αSMA expression in CD90^+^/cKit^−^ cardiac fibroblasts^44^.

Constituting a pivotal mechanism in the activation of the fibrogenic response and its strong association to cardiac fibrosis^45^, we thus tested Rhein in the setting of TGFβ-mediated FMT as proof-of-concept. A key finding of this work was that Rhein mitigated both TGFβ1-induced expression as well as protein abundance of αSMA in a dose-dependent matter. Analysis of its canonical pathway demonstrated impeded transcription of pro-fibrotic genes, obstructed levels of SMAD2 phosphorylation and reduction of TGFβRI transcription and abundance, pointing towards Rhein-interceded abrogation of the TGFβ/SMAD-signaling. This was in line with observations made in other tissues^15,26^. SMAD7 as endogenous TGFβ/SMAD-signaling inhibitor, associates to TGFβRI, labeling it for recruitment of ubiquitin ligases and subsequent proteasomal degradation^46^. Here we reported, that Rhein led to increased transcription-independent SMAD7 and SMAD-specific E3 ubiquitin ligase SMURF2 protein abundance. In contrast, TGFβ receptor regulated SMAD2/3 protein abundances were not affected by Rhein. This would implicate an interference of Rhein on TGFβ signaling due to increased inhibition. Interestingly, also SMAD7 is constantly subjected to ubiquitination-mediated proteasome degradation and depletion is counteracted by competitive acetylation of lysine residues^47–49^. In the present study, Rhein was already demonstrated to effectively diminish HDAC activity and promoting the posttranslational stabilization of p53. At this point, a second piece of evidence was determined, as HDAC inhibition using the reference inhibitor SB increased SMAD7 stability comparably to Rhein. Although, these findings increasingly support Rhein as valid HDAC inhibitor and implicate its effect to be HDAC-dependent, the present studies still harbor limitations. The functional validation of cell cycle control or the final proof of SMAD7’s acetylation state after Rhein treatment in analogy to p53-acetylation would be required. Due to the inaccessibility of antibodies directed against acetylated SMAD7, this question could not be answered during this project.

Finally, the present study delivered novel functional evidence for Rhein as anti-fibrotic agent *in vitro* in the context of cardiac fibrosis, as demonstrated by FPCL contraction assays. Rhein administration dose-dependently inhibited TGFβ1-stimulated, FMT-associated collagen contraction correlating to decreased levels of αSMA, displaying similarly proportional effect sizes in the context of Rhein dose and TGFβ1. Another noteworthy observation constitutes that Rhein treated FPCLs scored diminished basal contraction levels in comparison to unstimulated controls. This may be indicative of that Rhein administration even overcomes culture condition-driven basal FMT observed in unstimulated cells, thus, emphasizing its potency.

Collectively, these data indicate a role of HDAC activity and acetylation states in the phenotypic transition of fibroblasts to myofibroblasts, both under pathological hypoxia and increased TGFβ1 presence. In this study, we identified Rhein as a novel potent inhibitor of HDAC activity with anti-fibrotic potency. Pathophysiologically, these data clearly ascribe Rhein a pharmacological action to abrogate myofibroblast radiation and propagation, by modulating released paracrine and autocrine factors and decreasing their proliferative capacity. In the future, translation into long-term *in vivo* studies should be considered, investigating the potential prolongation of the life span in individuals at risk of premature and accelerated heart failure due to cardiac fibrosis. Thus, repurposing of Rhein, as readily available drug with approved safety, could constitute a promising potential therapeutic approach in the supplemental and protective intervention of cardiac fibrosis, preventing its propagation in high risk patients and aiding the preservation of cardiac function.

## Methods

### HCF-v culture and treatment with recombinant TGFβ1

Primary HCF-v of 5 different healthy donors (31–57 y, caucasian, 2 female and 3 male) were purchased (Lonza, Basel, Switzerland; Promocell, Heidelberg, Germany) and cultured in FGM-3 medium (Lonza, Basel, Switzerland) in 6-well plates (37 °C, 5% CO_2_). Sub-confluent HCF-v were serum-starved and treated with 10 ng/ml human recombinant TGFβ1 (Peprotech, Hamburg, Germany) alone or in combination with 15 or 35 µM Rhein for 24 h or 30 min as indicated or left untreated for control.

### Chronic hypoxia treatment

HCF-v were seeded in 145 mm dishes. After 16 h, cells were subjected to the hypoxic workstation (Xvivo System (Biospherix, Parish, NY, USA) set to 0.5% O_2_, 5% CO_2_, 94.5% N_2_ and 37 °C) with N_2_-pre-gassed DMEM (10% FCS, 1% glutamine, 1% antibiotic/antimycotic). After 60 h, sub-confluent cells were washed (1x PBS) and serum-starved in pre-nitrogenated serum-free media (DMEM, 1% glutamine, 1% antibiotic/antimycotic) for 36 h for secretome collection. For normoxic control, cells were treated in parallel in a second incubator of the hypoxic workstation (21% O_2_, 74% N_2_, 5% CO_2_ and 37 °C). For Rhein treatment, 35 µM Rhein was added during normoxic or hypoxic treatment for a total incubation time of 96 h and refreshed with every change of the culture medium (every 30 h). Collected supernatants were filtered through a 0.8 µM syringe filter and immediately stored at −80 °C before subjected to secretome analysis. The cell pellets were washed twice (1x PBS) and either dry-frozen or directly processed for further measurements.

### Fibroblast-populated collagen lattice (FPCL) contraction assay

Collagen media was prepared on ice by mixing 1 part of rat-tail collagen type I (4 mg/ml CellSystems, Troisdorf, Germany) with 3 parts (v/v) of growth media. For gel polymerization 1:25 NaOH (1 M) (v/v) was added to the collagen media and immediately mixed with HCF-v suspension (final concentrations: 1 mg/ml collagen I; 300,000 cells/ml). Collagen/cell-mixtures were transferred to a 24-well plate solidified in a humidified incubator (15 min, 37 °C). Polymerized lattices were transferred in triplicates to a 6-well plate containing serum-free media and treated with 10 ng/ml TGFβ1 and/or 15 µM or 35 µM Rhein for 5 days. Overhead pictures were recorded at day d0 (before treatment) and d5 using the ChemiDoc System (BioRad, Munich, Germany) and surface areas were determined manually using ImageJ software (National Institute of Mental Health, Bethesda, Maryland, USA). Relative contraction was calculated by dividing final area (d5) by starting area (d0).

### Transcriptome analyses

RNA extraction (QIAGEN, Hilden, Germany) of snap frozen cells were performed as described^50^. For quality control and quantification aliquots (1 µl) were analyzed with the RNA 6000 nano Kit on a Bioanalyzer 2100 (Agilent) and Nanodrop (Thermofisher Scientific). For transcriptome analysis, 150 ng RNA was processed as previously described^50^ and hybridized on Affymetrix Human Transcriptome Array 2.0 according to the manufacturer’s staining and scanning protocol. Data were analyzed using Expression console (Thermofisher Scientific, Darmstadt, Germany) and Transcriptome Analysis Console (TAC) software version 4.0 (Thermofisher Scientific, Darmstadt, Germany) (1.5-fold, p-value 0.05) as previously described^51^. Full datasets are available under GSE136039 on https://www.ncbi.nlm.nih.gov/geo. Further bioinformatic analysis was performed using the knowledge-based Ingenuity Pathway Analysis (IPA) (release summer 2018 (QIAGEN, Hilden, Germany)) QIAGEN Inc., https://www.qiagenbioinformatics.com/products/ingenuity-pathway-analysis ^52^.

### Realtime-qPCR

RNA was extracted using the Maxwell 16 LEV automated system and the LEV simplyRNA Purification Kit (Promega, Madison, USA), reverse transcribed using the Go Script Reverse Transcription System (Promega, Madison, USA) and Realtime-qPCRs were performed using the GoTaq qPCR master mix (Promega, Madison, USA) according to the manufacturer’s instructions and run on the StepOnePlus qPCR System (Thermofisher Scientific, Darmstadt, Germany) using primers for: *GLUT1* (5′-CCTGTCTCTTCCTACCCAACC-3′/5′-GCAGGAGTGTCCGTGTCTTC-3′), *SMAD7* (5′-GGCATTCGTCGGAAGTCAAG-3′/5′-AGAGTCGGCTAAGGTGATGG-3′), *EIF4A2* (5′-TGTGCAACAAGTGTCTTTGGTT-3′/5′-CACCTTTCCTCCCAAATCGAC-3′), *YHWAZ* (5′-TCTGGCTCCACTCAGTGTCT-3′/5′-CTGTGGGATGCAAGCAAAGG-3′). Target gene expression was normalized on *EIF4A2* and *YHWAZ* signals.

### Western blot analysis

For protein extraction, cells were lysed in lysis buffer (30 mM Tris‐HCl (pH 7.5), 1 mM EDTA, 150 mM NaCl, 0.5% Triton X‐100, 0.5% Na‐deoxycholate) containing protease and phosphatase inhibitors. Concentration was estimated using the BCA Protein Assay Kit (Pierce, Rockford, USA) according to the manufacturer’s prescription and 20 µg of lysate were loaded onto SDS-PAGE gels for separation. Proteins were blotted to polyvinylidene fluoride (PVDF) membranes via Mini PROTEAN Tetra System (BioRad, Munich, Germany) and transferred proteins were targeted using antibodies directed against HIF1α (Novus Biologicals, Littleton, USA), αSMA (Sigma‐Aldrich, Darmstadt, Germany), SMAD7 (R&D Systems, Wiesbaden, Germany), phospho(Ser465/Ser467)-SMAD2, Acetyl(Lys382)-p53, p53, p21, γ-tubulin and GAPDH (Cell Signaling Technology, Danvers, USA). Following incubation with HRP-conjugated antibodies against mouse or rabbit (Cell Signaling Technology, Danvers, USA), signals were detected using Immobilon Western Chemiluminescent HRP Substrate (Merck Chemicals GmbH, Darmstadt, Germany) and the VersaDoc MP4000 system (BioRad, Munich, Germany). Bands were analyzed using ImageLab 5.2 software (BioRad, Munich, Germany) and intensities were normalized to GAPDH or γ-tubulin as loading control.

### Proteomic profiling of secretome

Pre-filtered secretomes were centrifuged (45 min, 85,000 × g, 4 °C) prior concentration processing by centrifugation in Amicon Ultra-15 Ultracel column tubes (Merck Millipore, Burlington, USA) with an exclusion of proteins <3 kDa. Protein profiling of the secretomes was performed using LC-MS instrumentation consisting of an Ultimate 3000 separation liquid chromatography system (Thermofisher Scientific, Germering, Germany) combined with an EASY-spray ion source and Orbitrap Fusion Lumos Tribrid mass spectrometer (Thermofisher Scientific) as previously described^53^. Peptides were trapped on an Acclaim PepMap C18-LC-column (ID: 75 μm, 2 cm length; Thermofisher Scientific) and separated via EASY-Spray C18 column (ES802; ID: 75 μm, 25 cm length; Thermofisher Scientific). Each LC-MS run lasted 150 min and MS data were acquired with both data-dependent (DDA) and data-independent (DIA, 34 windows) MS/MS scan approaches. DDA runs were analyzed using Proteome Discoverer 2.2 software (Thermofisher Scientific) and Sequest HT search (trypsin digestion, max. 2 miscleavages, 5–144 peptide length, max. 10 peptides per spectrum, carbamidomethylation as static and N-terminal acetylation/methionine oxidation as dynamic modifications) against the SwissProt FASTA database (Homo sapiens (TaxID = 9606, version 2018-05-25)). Percolator node-based peptide-spectrum-match (PSM) analysis was restricted to q-values with 0.01 (strict) and 0.05 (relaxed) false discovery rate (FDR). Proteins were filtered using parsimony principle set to 0.01/0.05 (strict/relaxed) FDRs. For quantification, DIA runs were analyzed via Spectronaut PulsarX 11.01 software (Biognosys, Zürich, Switzerland) set to standard parameter settings and using a self-performed spectral library based on DDA runs. For retention time alignment, the secretomes were spiked with indexed Retention Time (iRT) standard. The mass spectrometry proteomics data have been deposited to the ProteomeXchange Consortium via the PRIDE^54^ partner repository with the dataset identifier PXD016731.

### HDAC I/II activity assay

Cell lysates were prepared using 1% NP‐40 (in 1x PBS) and 5 µg of protein was incubated with HDAC class I/II substrate (HDAC-Glo I/II Assay and Screening System, Promega, Madison, IA, USA) for 45 min (RT), before luminescence was determined. To study inhibitory effects, 35 µM Rhein or 1 mM sodium butyrate (SB) as reference HDAC inhibitor was applied directly to the lysates (30 min pre-incubation (RT) and during the assay run).

### Statistical analysis

Statistical analysis was performed with GraphPad Prism 7.04 (GraphPad Software Inc., La Jolla, USA). To confirm significant differences, One-way-ANOVA with Sidak’s multiple comparison test was used as detailed in the legends of the figures. Values are presented as means with standard deviation (SD)^55^.

## Supplementary information

Dataset 2.

Dataset 1.
