# Supplementary material for: Rhein, a novel Histone Deacetylase (HDAC) inhibitor with antifibrotic potency in human myocardial fibrosis
Source: Sci Rep. 2020 Mar 17;10:4888. doi: 10.1038/s41598-020-61886-3 (PMC7078222; doi:10.1038/s41598-020-61886-3)
Supplement: Supplementary file 1 — Dataset 2. [file 41598_2020_61886_MOESM1_ESM.pdf]

Supporting Information

Rhein, a novel Histone Deacetylase (HDAC) inhibitor with antifibrotic potency in human myocardial fibrosis

David Monteiro Barbosa, Pia Fahlbusch, Daniella Herzfeld de Wiza, Sylvia Jacob, Ulrike Kettel, Hadi Al-Hasani, Martina Krüger, D. Margriet Ouwens, Sonja Hartwig, Stefan Lehr, Jorg Kotzka, Birgit Knebel

Supplementary Figure 1

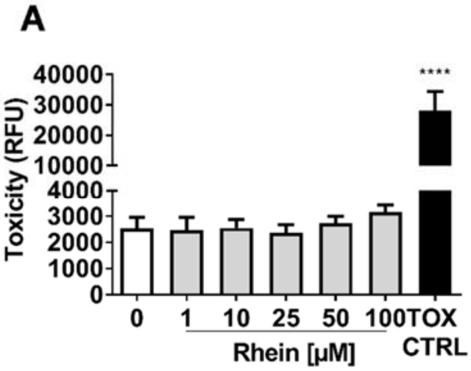

Rhein is not toxic in HCF-v up to 100µM. HCF-v were treated with a dose-range of 1-100µM Rhein for 24h or with lysis reagent for 15min as maximum toxicity control (tox control). Cytotoxicity was measured using CellTox Green Assay (Promega) and is given in relative fluorescent units (RFU) (n=4). One-way-ANOVA with post-hoc Sidak´s multiple comparison, ****p<0.001 as indicated.

Supplementary Figure 2

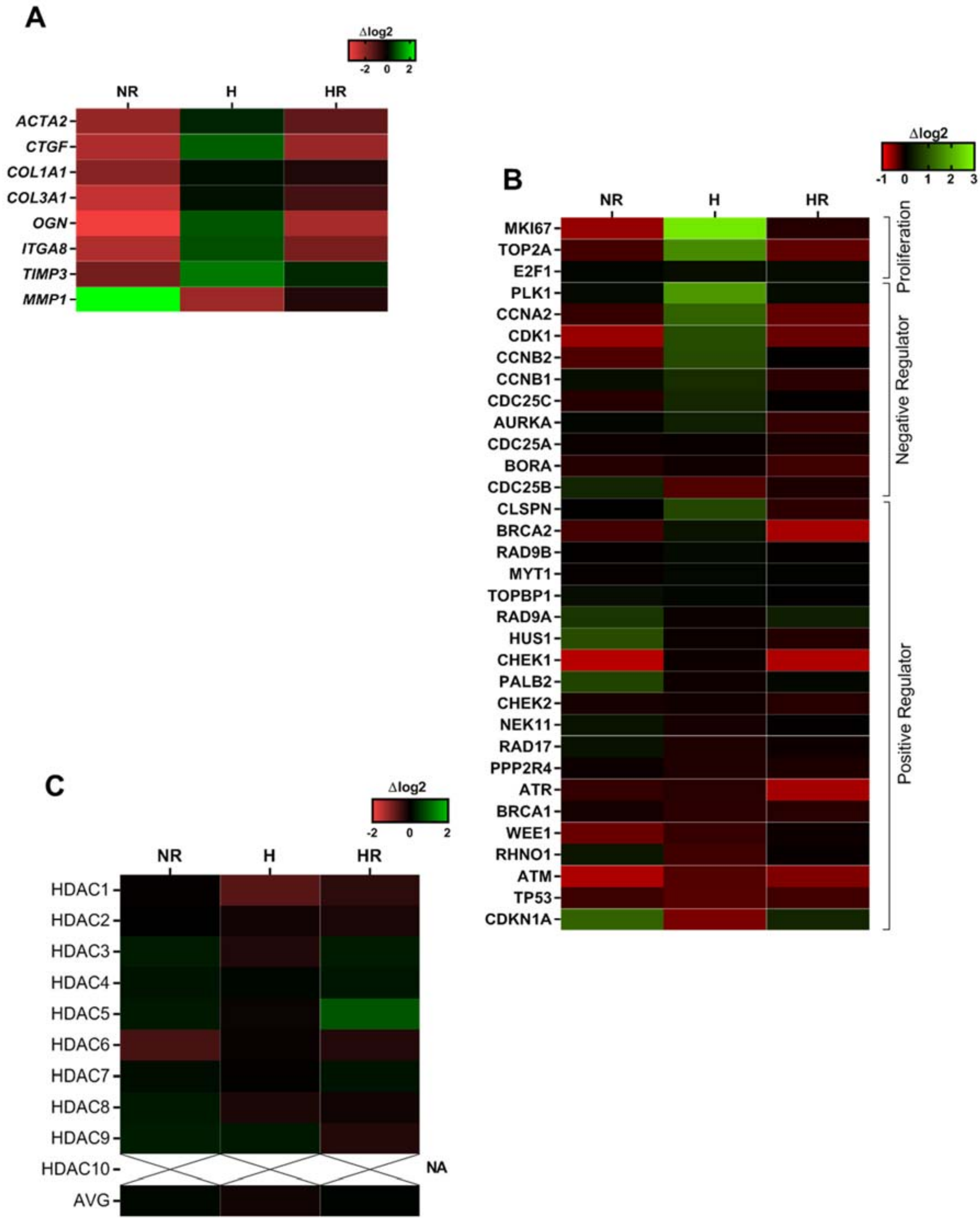

Effects of Rhein on transcriptomic expression of pro-fibrotic, G2/M cell cycle checkpoint and HDAC genes (n=4). **A** Analysis of selected pro-fibrotic FMT markers. **B** Analysis of proliferation markers and positive and negative regulators of G2/M phase. **C** Analysis of HDAC gene family members. The heat maps represent transcriptional expression scaled as Log_2 in relation to normoxic control as $\Delta log_2 = Log_2(x)-Log_2(Normoxia)$ with x=NR (Normoxia+Rhein), H (Hypoxia), HR: Hypoxia+Rhein; $\Delta log_2 \geq 0.58 \triangleq 1.5$-fold (linear).

Supplementary Figure 3

Cell Cycle G1/S Checkpoint Regulation

H vs N

no
molecules of this pathway
in comparision H vs N

HR vs NR

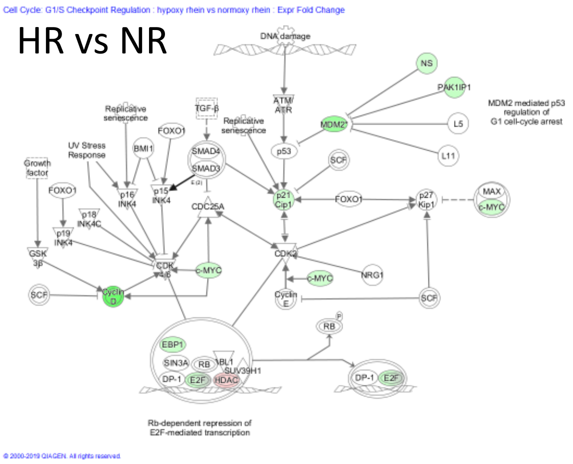

N vs NR

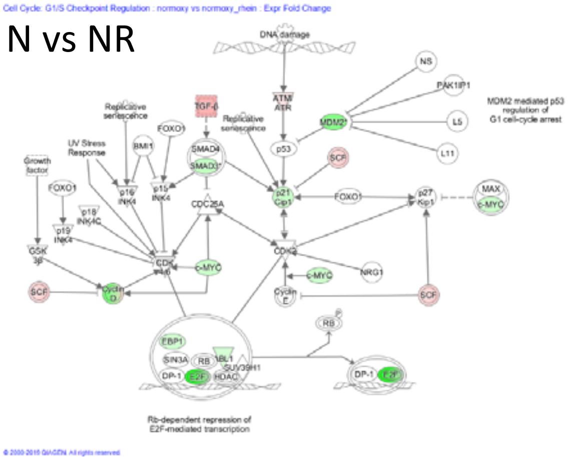

H vs HR

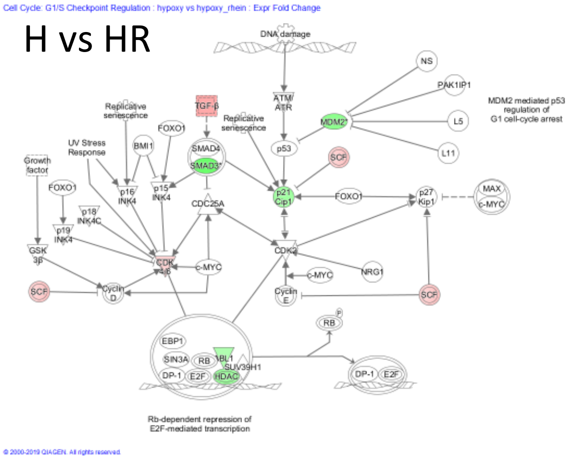

Symbol	Entrez Gene ID for Human	H vs N Expr Fold Change	H vs N Expr p-value	H vs N Expr False Discovery Rate (q-value)	HR vs NR Expr Fold Change	HR vs NR Expr p-value	HR vs NR Expr False Discovery Rate (q-value)	N vs NR Expr Fold Change	N vs NR Expr p-value	N vs NR Expr False Discovery Rate (q-value)	H vs NR Expr Fold Change	H vs NR Expr p-value	H vs NR Expr False Discovery Rate (q-value)
**ABL1**	25							-1,680	4,36E-02	4,24E-01	-1,560	3,81E-02	1,00E00
**ATM**	472							1,600	3,75E-02	3,91E-01			
**CCND1**	595				-5,120	6,00E-04	4,74E-02	-5,340	1,90E-03	6,47E-02			
**CCND2**	894							2,290	1,67E-02	2,47E-01			
**CDK6**	1021										1,650	2,79E-02	1,00E00
**CDKN1A**	1026				-1,650	2,80E-03	1,03E-01	-2,220	3,73E-05	6,00E-03	-1,880	3,40E-03	7,15E-01
**E2F3**	1871				-1,760	2,40E-03	9,54E-02	-1,700	3,70E-03	9,70E-02			
**E2F4**	1874							-1,740	1,90E-03	6,29E-02			
**E2F5**	1875				-1,640	8,10E-03	1,75E-01						
**E2F6**	1876				-1,570	1,20E-03	6,61E-02						
**E2F7**	144455							-12,610	1,00E-02	1,80E-01			
**FBXL5**	26234							2,090	2,90E-03	8,28E-02	1,580	2,50E-03	6,47E-01
**GNL3**	26354				-1,540	5,20E-03	1,42E-01						
**H vs HR**													
**HDAC5**	10014				1,570	4,64E-02	3,49E-01				-1,980	2,10E-03	6,24E-01
**MDM2**	4193				-3,420	1,37E-05	6,50E-03	-4,880	8,72E-07	7,00E-04	-2,240	4,90E-03	7,55E-01
**MYC**	4609				-1,620	3,63E-02	3,22E-01	-2,080	5,70E-03	1,27E-01			
**N vs NR**													
**PA2G4**	5036				-1,850	5,39E-06	4,00E-03	-1,880	2,19E-05	4,60E-03			
**PAK1IP1**	55003				-1,990	3,90E-03	1,22E-01						
**SKP2**	6502							2,880	1,10E-03	4,66E-02	2,290	6,70E-03	7,93E-01
**SMAD3**	4088							-2,380	5,30E-03	1,21E-01	-2,930	1,50E-03	5,73E-01
**TGFB3**	7043							4,470	3,80E-03	9,82E-02	3,210	7,10E-03	7,93E-01

Cell Cycle G2/M DNA Damage Checkpoint Regulation

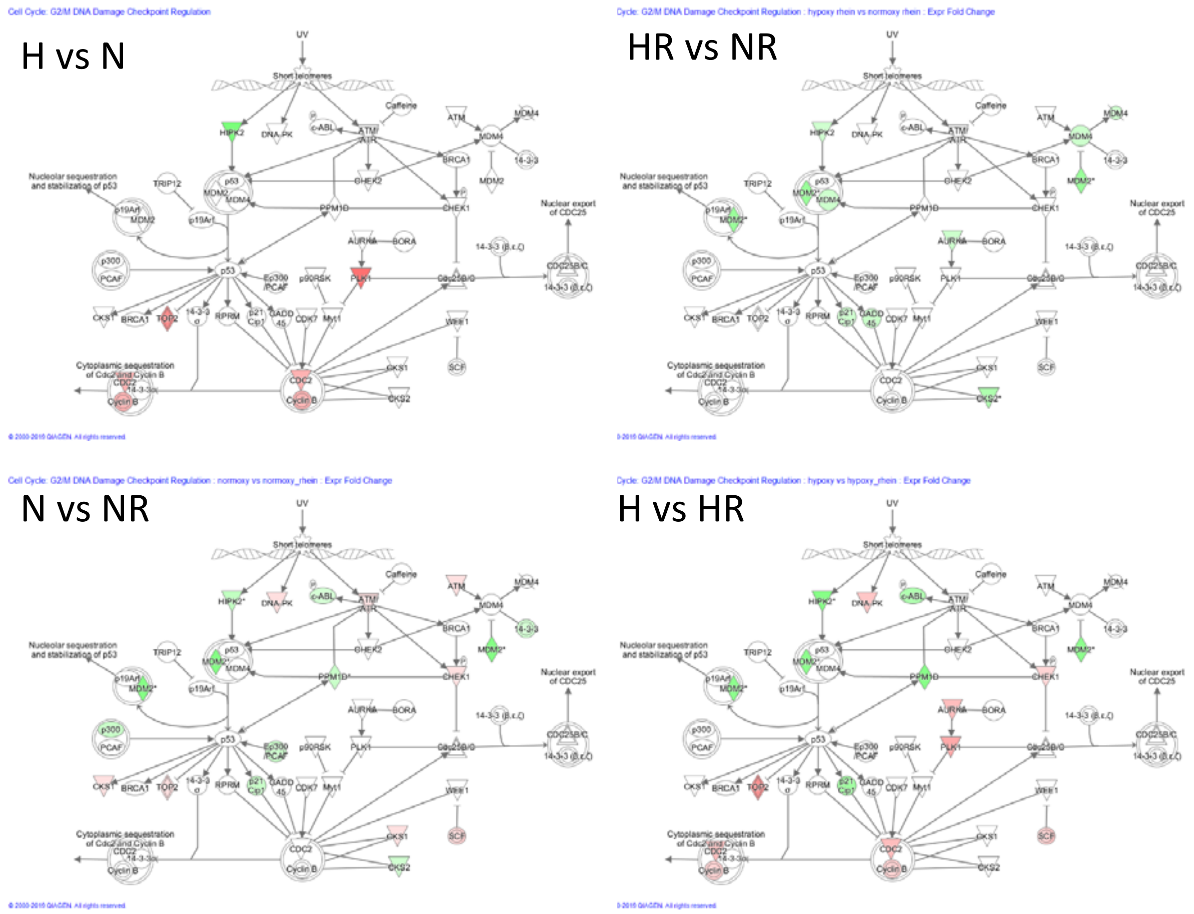

Symbol	Entrez Gene ID for Human	H vs N			HR vs NR			N vs NR			H vs NR		
		Expr Fold Change	Expr p-value	Expr False Discovery Rate (q-value)	Expr Fold Change	Expr p-value	Expr False Discovery Rate (q-value)	Expr Fold Change	Expr p-value	Expr False Discovery Rate (q-value)	Expr Fold Change	Expr p-value	Expr False Discovery Rate (q-value)
**ABL1**	25							-1,680	4,36E-02	4,24E-01	-1,560	3,81E-02	1,00E00
**ATM**	472							1,600	3,75E-02	3,91E-01			
**AURKA**	6790				-1,720	4,40E-02	3,42E-01				2,440	1,80E-03	5,86E-01
**CCNB1**	891										1,620	5,70E-03	7,85E-01
**CCNB2**	9133	1,820	1,03E-02	8,13E-01							1,830	1,90E-03	6,09E-01
**CDK1**	983	1,850	4,07E-02	8,13E-01							2,460	1,10E-03	5,73E-01
**CDKN1A**	1026				-1,650	2,80E-03	1,03E-01	-2,220	3,73E-05	6,00E-03	-1,880	3,40E-03	7,15E-01
**CHEK1**	1111							1,660	3,50E-03	9,41E-02	1,560	1,90E-02	9,70E-01
**CKS1B**	1163							1,770	1,69E-02	2,49E-01			
**CKS2**	1164				-2,700	1,30E-03	6,81E-02	-1,820	3,73E-02	3,90E-01			
**EP300**	2033							-2,160	4,11E-05	6,40E-03			
**FBXL5**	26234							2,090	2,90E-03	8,28E-02	1,580	2,50E-03	6,47E-01
**GADD45A**	1647				-1,870	2,40E-02	2,77E-01						
**HIPK2**	28996	-1,990	3,29E-02	8,13E-01	-1,600	1,27E-02	2,12E-01	-2,470	3,72E-02	3,89E-01	-2,360	2,23E-02	9,90E-01
**MDM2**	4193				-3,420	1,37E-05	6,50E-03	-4,880	8,72E-07	7,00E-04	-2,240	4,90E-03	7,55E-01
**MDM4**	4194				-1,690	1,50E-03	7,57E-02						
**PLK1**	5347	3,450	1,50E-03	8,13E-01							3,180	4,00E-04	5,05E-01
**PPM1D**	8493							-2,160	1,80E-03	6,21E-02	-2,370	4,10E-03	7,42E-01
**PRKDC**	5591							1,850	1,21E-02	2,02E-01	2,090	1,07E-02	8,85E-01
**SKP2**	6502							2,880	1,10E-03	4,66E-02	2,290	6,70E-03	7,93E-01
**TOP2A**	7153	2,730	3,31E-02	8,13E-01							4,020	2,30E-03	6,46E-01
**TOP2B**	7155							1,710	7,80E-03	1,55E-01			
**YWHAG**	7532							-1,690	2,80E-03	8,15E-02			

Gene expression data (1.5 fold-change; p<0.05) were analyzed using the knowledge-based Ingenuity Pathway Analysis (IPA) (release summer 2018 (QIAGEN, Hilden, Germany)). The networks were generated through the use of IPA (QIAGEN Inc., https://www.qiagenbioinformatics.com/products/ingenuity-pathway-analysis).

Pathway Molecules

All Pathway molecules were selected through the use of IPA (QIAGEN Inc., https://www.qiagenbioinformatics.com/products/ingenuity-pathway-analysis).

G1/S Checkpoint Regulation: ABL1, ATM/ATR, BMI1, CDC25A, CDK2, CDK4/6, CDKN1A, CDKN1B, CDKN2A, CDKN2B, CDKN2C, CDKN2D, Cyclin D, Cyclin E, DNA damage, E2f, E2f-Tfdp1, FOXO1, GNL3, GSK3B, Hdac, MAX, Max-Myc, MDM2, MYC, NRG1, PA2G4, PAK1IP1, Rb, Rb-E2F transcription repression, Replicative senescence of cells, RPL11, RPL5, Scf, SIN3A, SMAD3, Smad3-Smad4, SMAD4, SUV39H1, TFDP1, Tgf beta, TP53.

G2/M DNA Damage Checkpoint Regulation : 14-3-3, 14-3-3 (β,ε,ζ), 14-3-3-Cdc25, ABL1, ATM, ATM/ATR, AURKA, BORA, BRCA1, Cdc2-CyclinB-Sfn, Cdc25B/C, CDK1, CDK1-Cyclin B, CDK7, CDKN1A, CDKN2A, Cdkn2a-Mdm2, CHEK1, CHEK2, CKS1B, CKS2, Cyclin B, EP300, Ep300/Pcaf, GADD45A, HIPK2, KAT2B, MDM2, Mdm2-Tp53-Mdm4, MDM4, PKMYT1, PLK1, PPM1D, PRKDC, RPRM, RPS6KA1, Scf, SFN, Top2, TP53, TRIP12, WEE1

Conditions: N: Normoxia, H: Hypoxia, NR: Normoxia+Rhein, HR: Hypoxia+Rhein

Supplementary Figure 4

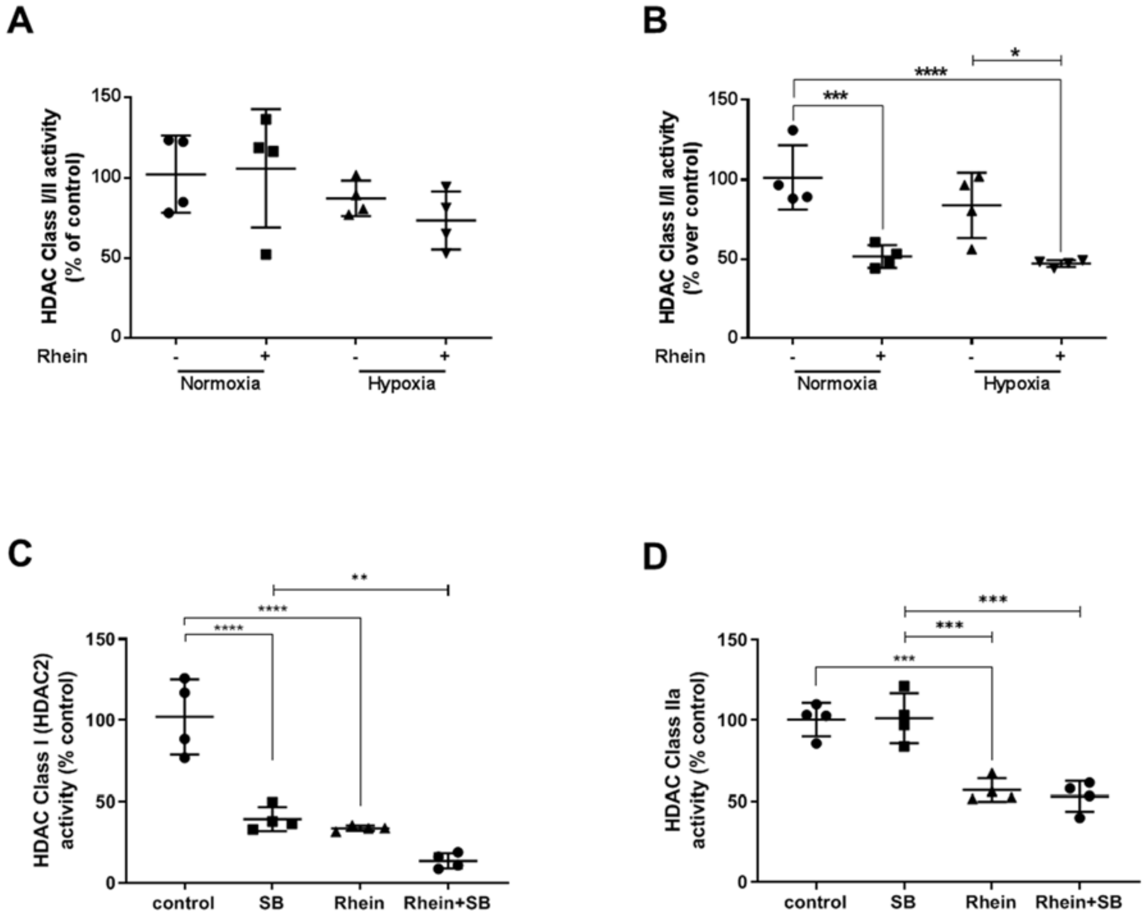

Rhein reversibly inhibits HDAC activity. **A** HCF-v that underwent the hypoxic or normoxic protocol with or without 96h Rhein treatment were washed twice and lysed in 0.1% Triton-X/PBS. Measurement of HDAC Class I/II activity in these lysates did not show any differential effects (n=4). **B** HDAC Class I/II activity in the very same lysates was significantly decreased, if the lysates were incubated with 35µM Rhein directly for 30min prior to the assay and during assay performance. Rhein efficiently inhibits both HDAC Class I and IIa (n=4). **C,D** Normal HCF-v lysates were treated with 1mM sodium butyrate, 35µM Rhein or with both in combination and subjected to HDAC Class I (HDAC2) (**C**) or HDAC Class IIa (**D**) activity assays, respectively (n=4). All data are presented as mean±SD. One-way-ANOVA with post-hoc Sidak´s multiple comparison, *p<0.05, **p<0.01, ***p<0.001, ****p<0.001 as indicated.

Supplementary Figure 5

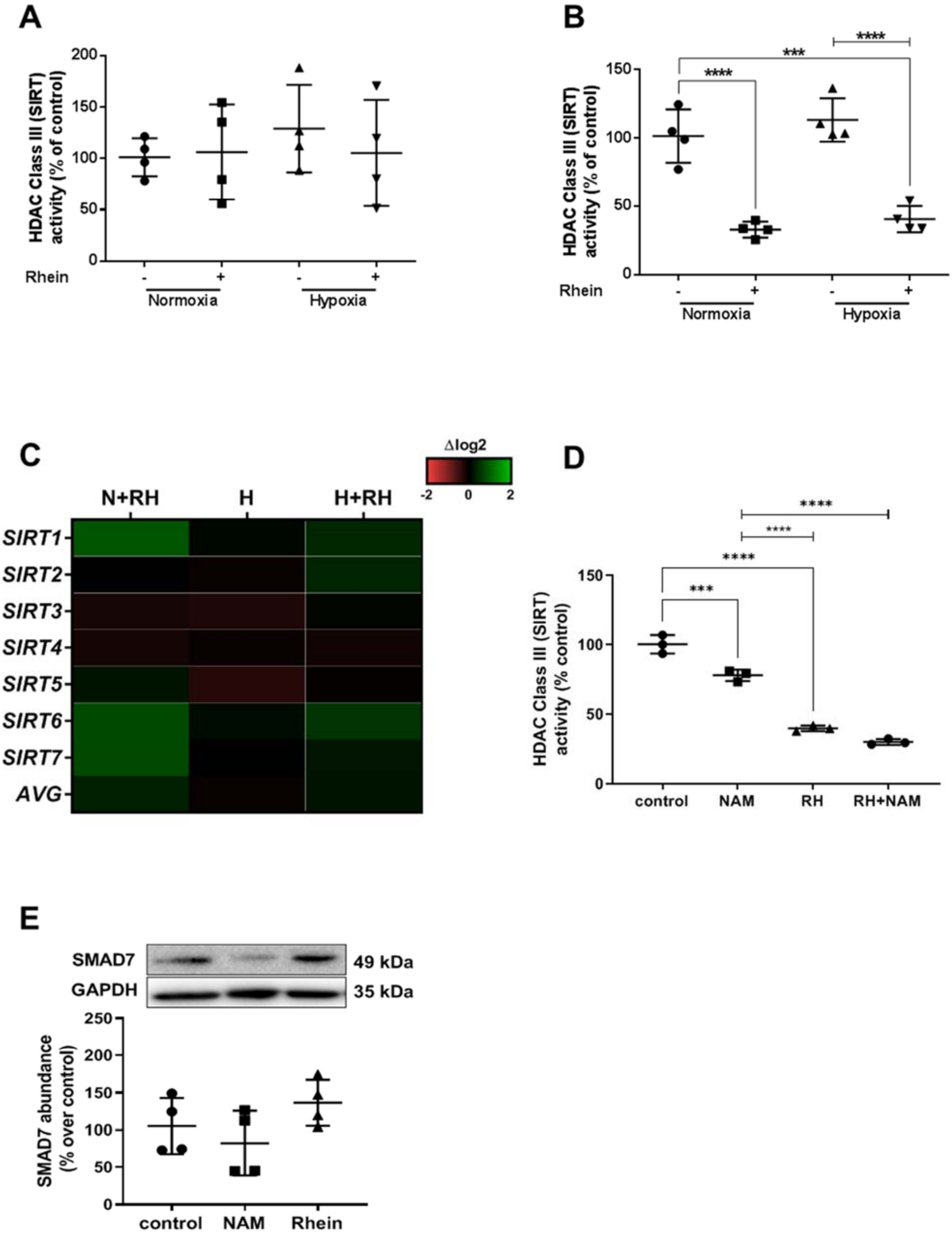

Rhein reversibly inhibits SIRT activity. **A** HDAC Class III (SIRT) activity was measured in protein lysates of HCF-v that were treated with Rhein and underwent the hypoxic or normoxic

protocol showing no differential effects (n=4). **B** HDAC Class III (SIRT) activity in the very same lysates after addition of 35µM Rhein for 1h and during assay performance however, was significantly decreased (n=4). **C** Analysis of SIRT gene family members. The heat maps represent transcriptional expression scaled as Log_2 in relation to normoxic control as $\Delta log_2 = Log_2(x) - Log_2(Normoxia)$ with x=NR (Normoxia+Rhein), H (Hypoxia), HR: Hypoxia+Rhein; $\Delta log_2 \geq 0.58 \triangleq 1.5$-fold (linear). **D** Rhein strongly inhibits SIRT activity. Normal HCF-v lysates were treated with 10mM nicotinamide (NAM; SIRT inhibitor), 35µM Rhein or with both in combination and subjected to HDAC Class III (SIRT) activity assay (n=3). **E** SIRT inhibition does not affect SMAD7 stabilization. Representative blot and quantification showing different effect of Rhein and NAM treatment (n=4). All data are presented as mean±SD. One-way-ANOVA with post-hoc Sidak´s multiple comparison, ***p<0.001, ****p<0.001 as indicated.

Supplementary Figure 6

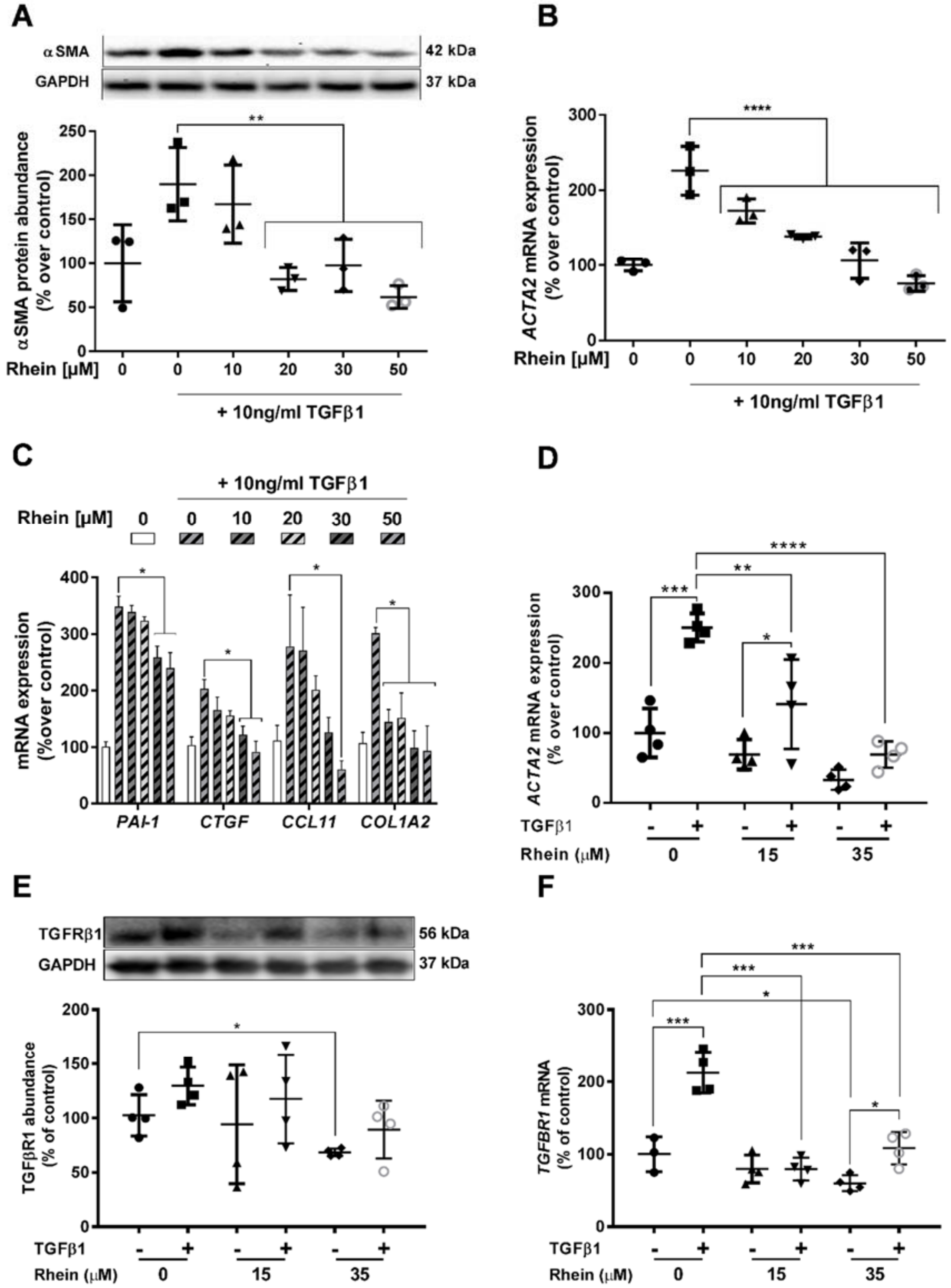

Dose-dependent effects of Rhein on TGFβ1-mediated HCF-v stimulation. **A** Representative blot and quantification showing αSMA protein abundance in HCF-v treated with 10ng/ml TGFβ1 and 10-50µM Rhein in parallel (n=3). **B** Graph showing TGFβ1-mediated *ACTA2* gene expression decreases in response to Rhein (n=3). **C** Rhein dose-response in other pro-fibrotic genes. **D** *ACTA2* expression levels showing Rhein-mediated inhibition of TGFβ1-stimulated

activation (n=4). **E** Representative blot and quantification showing TGFβR1 protein decrease in Rhein treated cells (n=4). **F** Expression analysis of *TGFBR1* showing increased transcription after TGFβ1 and Rhein-mediated abrogation (n=4). All data are presented as mean±SD. One-way-ANOVA with post-hoc Sidak´s multiple comparison, *$p<0.05$, **$p<0.01$, ***$p<0.001$, ****$p<0.001$ as indicated.

Supplementary Figure 7

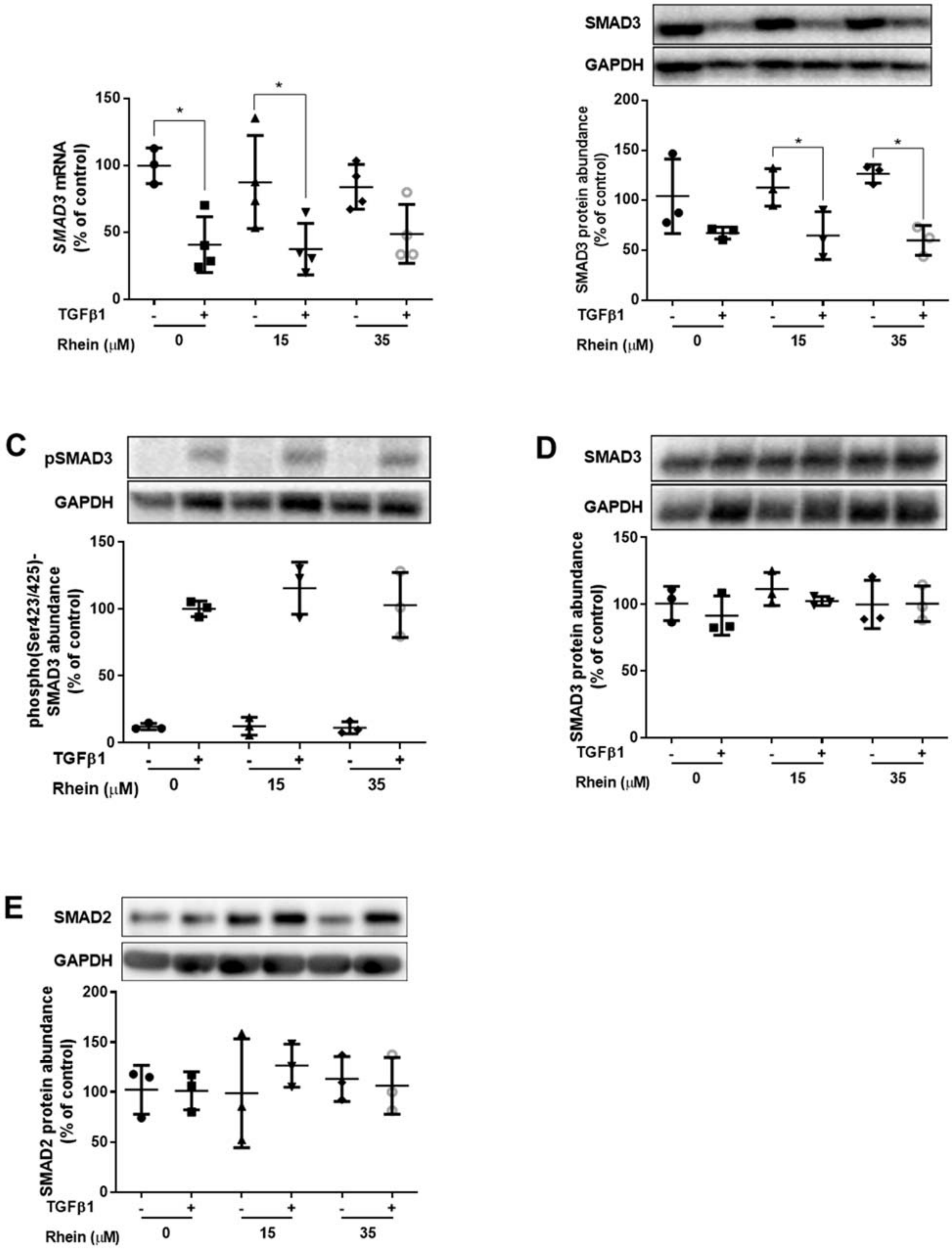

A,B HCF-v were treated with 15µM or 35µM Rhein with or without recombinant TGFβ1 for 24h (n=3-4). Graphs showing no Rhein-mediated effect on *SMAD3* mRNA (A) and protein levels (B) and no modulation of TGFβ1-stimulated negative feedback loop (mRNA and protein degradation). **C,D,E** HCF-v were treated with 15µM or 35µM Rhein for 24h before challenge for 30min with or without recombinant TGFβ1 administration (n=3-4). Graphs showing that Rhein does not modulate TGFβ1-mediated SMAD3 activation (C), total SMAD3 (D) and total SMAD2 (E) abundance within 30min. All data are presented as mean±SD. One-way-ANOVA with post-hoc Sidak´s multiple comparison, *p<0.05 as indicated.

Supplementary Figure 8

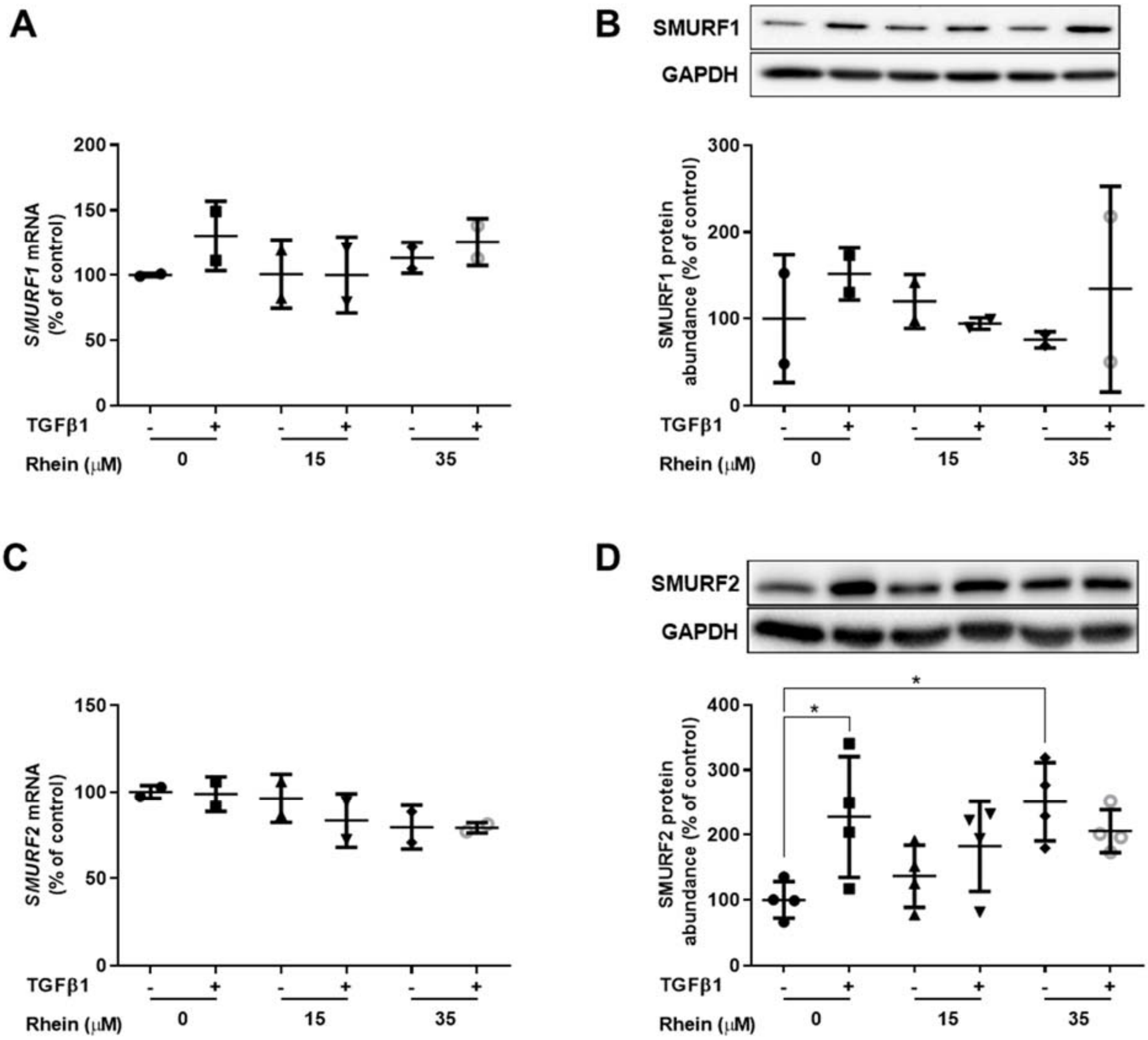

Rhein posttranslationally increases SMAD-specific E3 ubiquitin ligase SMURF2, but not SMURF1 protein abundance. HCF-v were treated with 15μM or 35μM Rhein with or without recombinant TGFβ1 for 24h. Graphs showing that neither *SMURF1* mRNA (**A**) nor protein abundance (**B**) are affected by Rhein or TGFβ1 (n=2). **C** RT-qPCR of *SMURF2* mRNA showing no differential effects (n=2). **D** Representative Western blot and quantification displays TGFβ1- and Rhein-mediated increase of SMURF2 protein abundance (n=4). All data are presented as mean±SD. One-way-ANOVA with post-hoc Sidak´s multiple comparison, *p<0.05 as indicated.

Supplemental methods

Cell toxicity assay

To determine the working dose range of Rhein and inhibitors used in this study, the non-toxic concentrations were analyzed using the cell-based CellTox Green Cytotoxicity Assay (Promega, Madison, IA, USA) according to the instruction manual. The principle of the assay is based on a fluorescent DNA-binding dye that is excluded from viable cells, but preferentially enters non-viable cells due to their lack of membrane integrity. The resulting fluorescent signal is directly proportional to the stained cells and cytotoxicity. 10,000 HCF-v cells per well were seeded into black opaque 96-well cell culture plates in growth media and let adhere overnight. Cells were washed once with PBS and dose range of respective substance of interest was applied to the cells in serum-free media for 24h at 37°C in a humidified incubator (74% N_2, 21% O_2, 5% CO_2). For assaying, 100 µl of dye-containing assay buffer (1:1 to medium (v/v)) was added to the cells and incubated for 15min RT prior measurement of fluorescent signals at EEx = 485nm/EEm = 530nm using the Infinite 200 PRO microplate reader. A maximum toxicity control was employed by addition of 4 µl lysis reagent to the cells.

SIRT/ HDAC activity assays

Measurement of respective enzyme-activities in this study were carried out using bioluminescence-based enzyme-specific activity assays, the SIRT-Glo, HDAC2-Glo and HDAC IIa-Glo (Promega, Madison, USA), according to the manufacturer's protocol. Briefly, for all activity assays used, 5 µg of whole cell extracts as enzyme source were diluted in 50 µl of respective assay buffer and added to white-walled (luminescence) opaque 96-well plates. If applicable, to monitor whether Rhein has a modulating effect on respective enzyme activity, 5 µg of control cell lysates (24h serum-free media) were pre-incubated with 35µM Rhein or with specific inhibitor for 30 min. Luminescence was measured using the Infinite 200 PRO microplate reader (Tecan Group AG, Zurich, Switzerland). As reference for testing the influence of Rhein on SIRT or HDAC activity, 10mM nicotinamide (NAM) or 1mM sodium butyrate (SB), respectively, were used as comparative inhibitors.
